# The Relation between Effortful Control and Language Competence—A Small But Mighty Difference between First and Second Language Learners

**DOI:** 10.3389/fpsyg.2016.01015

**Published:** 2016-07-06

**Authors:** Karin Keller, Larissa M. Troesch, Sarah Loher, Alexander Grob

**Affiliations:** ^1^Department of Psychology, University of BaselBasel, Switzerland; ^2^School Psychology Services, Canton of Basel-StadtBasel, Switzerland

**Keywords:** effortful control, second language, language development, temperament, dual language learner

## Abstract

The present longitudinal study evaluates the effect of *effortful control* (EC) as a core dimension of temperament on early language competence. We assume that first and second language competence is influenced by EC, and that immigrant children with low EC are thus at risk of an unfavorable language development. The sample consisted of *n* = 351 dual language learners (DLLs) with an immigrant background and *n* = 78 monolingual children. Language competence was measured with a standardized language test at age 4.9 years and at age 6.3 years. EC was captured with the Child Behavior Questionnaire, completed by teachers. Results of regression analyses revealed a significant effect of EC on second language development. DLLs with lower EC were found to have not only lower language competence at the beginning and the end of kindergarten but also a less favorable language development. Comparisons between the effect of EC on first and second language provide evidence that EC plays a bigger role in subsequent second language competence compared to first language competence. Overall, the results emphasize the small yet significant role of EC in the second language development of DLLs.

## Introduction

In school as well as everyday situations children are expected to regulate their behavior in order to reach goals, to accomplish tasks, and to monitor their activities (e.g., Blair and Razza, 2007; Neuenschwander et al., 2012). Not only highly structured situations such as school lessons but also low-threshold learning situations early in ontogeny have been shown to require qualities such as attentiveness, adaptability, and high reactivity (Rothbart and Jones, 1998). These behaviors are related to *effortful control* (EC), describing a subordinate component of temperament (Rothbart and Rueda, 2005). Language development as an example of such a low-threshold learning situation has thus been revealed to be linked to temperament and EC in normatively developing monolingual children (e.g., Slomkowski et al., 1992). Little is known, however, about the relationship between EC and language competence in dual language learners (DLLs) who acquire the local language as a second language, usually after the age of 3 years (Winsler et al., 2014; Chen et al., 2015). Individual differences in the DLL acquisition trajectories have shown a considerable heterogeneity among DLLs (e.g., Kim et al., 2014). Moreover, these children have been shown to face additional challenges, including lower second language competence and a slower second language development, compared to monolinguals’ first language development (Dubowy et al., 2008; Caspar and Leyendecker, 2011; Hoff, 2013). Comparing monolinguals and DLLs also reveals that the context of language learning differs remarkably between both groups (e.g., familial background). First language competence is mostly acquired in familial contexts, characterized by small dyads. Children who learn the local language as second language, however, usually acquire and use their second language in the context of kindergarten, characterized by larger dyads, more distraction, and higher demands on the control of attention. Second language contexts thus both require and train competences such as the inhibition of irrelevant impulses or switching the focus of attention. The present study therefore assesses the relationship between EC and language competence in monolinguals and DLLs.

We aim at contributing to this rarely studied research topic of EC and its relation to language competence in DLLs by assessing EC as a predictor of the levels and trajectories of language competence in DLLs in kindergarten, both concurrently and longitudinally. In addition, we investigate whether the predictive power of EC on later language competence differs between DLLs and monolinguals.

### Effortful Control as a Component of Temperament: Definition and Developmental Trajectory

In the evaluation of reasons for individual differences in children’s behavior, temperament has been assigned a prominent role (Dixon et al., 2006). Temperament is defined as individual differences in emotional, motor, and attentional reactivity and self-regulation (Rothbart and Derryberry, 1981; Rothbart et al., 2003; Rothbart and Rueda, 2005). Regarding its factorial structure, three central factors of temperament have repeatedly been proposed (namely surgency, negative affectivity, and EC), suggesting a conceptual proximity to the big five personality dimensions extraversion, neuroticism, and conscientiousness in adulthood (Rothbart and Rueda, 2005). EC encompasses abilities such as the regulation of inhibitory control, attentional focusing, and perceptual sensitivity, thus allowing the resolution of conflict situations (Rothbart et al., 2003). In agreement with the literature, we define EC as efficiency of attentional control, including the inhibition of irrelevant actions and the generation of subdominant responses, and the ability to detect errors (Rothbart et al., 2003; Rothbart and Bates, 2006; Eisenberg et al., 2007).

Developmental trajectory of EC is shown to be protracted with developmental progression starting in early ontogeny (Rothbart et al., 2003) and continuing until early school years (Rothbart and Rueda, 2005; Eisenberg et al., 2011). The ability of adaptation and regulation of behavior is considered a prerequisite for the acquisition of cognitively more complex abilities, such as language competence and academic achievement, highlighting the importance of EC particularly around the transition to kindergarten and school (Wass et al., 2012; Merz et al., 2014).

Effortful control and its outcomes have been studied repeatedly in a variety of fields, in particular academic and pre-academic achievement, because classroom contexts pose increasing demands on children’s ability to control their behavior (e.g., Blair and Razza, 2007; Neuenschwander et al., 2012; Allan et al., 2014). In a recent meta-analysis, Allan et al. (2014) evaluated teacher ratings as being particularly valuable for assessing EC abilities in school situations. Similar findings were obtained by Blair and Razza (2007), who revealed teacher-rated EC being a longitudinal predictor for both mathematics and language competence. Neuenschwander et al. (2012) reported differential effects using parent-rated EC depending on specific outcomes: although EC predicted grades and learning behavior in the subsequent year, no longitudinal predictive power was found for EC regarding school achievement tests.

### Effortful Control as Predictor of Language Competence in Monolingual Children

Temperament and its relation to language and cognition has been studied extensively, mainly in monolingual children. The finding of differential interrelations between temperament and cognitive factors (verbal versus performance domains) has indicated that factors of temperament may also underlie individual differences in language development (for an overview, see Slomkowski et al., 1992). Empirical evidence indicated differential interrelations between language and the temperament factor of EC in receptive (e.g., Slomkowski et al., 1992; Morales et al., 2000), expressive (e.g., Blair and Razza, 2007; Usai et al., 2009), or both language modalities (e.g., Karrass and Braungart-Rieker, 2003).

Morales et al. (2000) assessed, among others, infant temperament (parental reports) and its relation to language competence in early ontogeny, in children aged 6–12 months. The authors propose that duration of orienting, inhibition, and distractibility may explain inter-individual differences in infant temperament, which in turn was assumed to be linked to subsequent language competence. The temperament dimension task orientation (assessed at 6 months of age) showed a positive longitudinal relationship with receptive language competence at 12 months whereas no interrelation was found with expressive language competence. Additionally, Karrass and Braungart-Rieker (2003) found significant positive bivariate relations between duration of orienting and language competence both concurrently assessed at 12 months, as well as between duration of orienting at the age of 12 months and subsequent language competence, assessed at 16 months. However, no significant effect was found in analyses controlling for the language level at age 12 months. In the early 1990s, Slomkowski et al. (1992) found a small yet significant positive correlation between EC (concentration, attention, and frustration level) and receptive and expressive language competence concurrently in 2-year-olds. This relation was also shown longitudinally with receptive language competence assessed 1 year thereafter. However, when the initial level of language was controlled, the effect of EC on language development disappeared. Blair and Razza (2007) reported positive concurrent links between teacher-rated EC and vocabulary in preschool children. However, no relation was found between parent-rated EC and vocabulary thus raising the perspective of the person rating children’s EC as an important influencing factor.

One explanation of the relation between EC and language has been presented by Bloom (1993). She postulated that a pool of underlying cognitive resources may be the basis of interpersonal expressions (Bloom, 1993; Dixon and Smith, 2000). As both emotion and language are viewed as being forms of interpersonal expressions and as having common functions, it has been suggested that they rely on this common pool of underlying cognitive resources. Consequently, greater emotional stability (as expressed in higher EC abilities) would lead to a facilitation of language acquisition because more attentional resources can be recruited for the process of language learning (for a thorough discussion, see Dixon and Smith, 2000). This approach resulted in the suggestion that the allocation of attentional resources is a central aspect in the relation between temperament factors and language development (e.g., Morales et al., 2000; Dixon et al., 2006). Dixon et al. (2006) thereby conclude that better abilities regarding the control and regulation of behavior and, in particular, of attentional resources, constitute better presuppositions for the acquisition of language competence. These better abilities allow a minimization of distractions and thus support language development indirectly. As a further explanation, Slomkowski et al. (1992) suggest that temperamental dimensions may affect children’s performance on mental tests. Moreover, the authors propose that temperamental dimensions may be reflected by cognitive and language styles.

Taken together, the literature review presented here indicates empirical evidence for the relation between EC and language components during early childhood. However, given the broad heterogeneity of assessed language outcomes in different reviewed studies it remains unclear whether there are specific aspects of language competence that are linked to EC most closely. Moreover, the majority of the existing studies have focused on concurrent or longitudinal interrelations *without* controlling for initial levels of performance (such as two reviewed examples of Morales et al., 2000 and Blair and Razza, 2007). Hence, the effect of EC on language *development* remains largely unexplored.

### Effortful Control and Second Language Competence

Only sparse evidence exists for the relation between EC and language in sequential DLLs. It has repeatedly been found that second language development is characterized by larger individual variability than the development of first language, most probably due to a greater diversity in familial as well as extra-familial contexts (Dubowy et al., 2008; Caspar and Leyendecker, 2011; Hoff, 2013; Kim et al., 2014; Winsler et al., 2014). As DLL children’s first (and mostly family) language is other than the local language, they all have limited to no exposition to the local language until they enter the formal school system (Becker, 2006). Thus, while monolingual children acquire their local language competence mostly in their familial context, the language acquisition context in DLLs is much more diverse and differs from monolinguals (Hoff, 2013). Despite the knowledge of these differences in the context of language development, there is a paucity of research about the influence of EC on second language development in immigrant children. There are few studies assessing EC in immigrant children. Notable exceptions are the recent studies by Bohlmann et al. (2015) and Chen et al. (2015). Similar to above-described findings in monolingual children, Chen et al. (2015) revealed a positive relation between EC and school achievement in immigrant children. These results may clarify the relation between EC and academic achievement as belonging to cognitive domains in a minority population of immigrants. However, they did not address the question of the influence of EC on language competence as a different aspect of cognitive domains. Moreover, even though they focused on first- versus second-generation immigrants, the language acquisition situation (monolinguals, parallel, or sequential bilinguals) was not focused on specifically. However, Bohlmann et al. (2015) assessed the interrelations between language competence and self-regulation in preschoolers with different language acquisition backgrounds in the US. Over three time points, the authors examined a potential bidirectionality between preschooler’s English language expressive vocabulary and self-regulation skills in monolinguals and DLLs. Results indicated that on the one hand, self-regulation skills seemed to be important for the development of expressive vocabulary. On the other hand, vocabulary was also evaluated as being important for the development of self-regulation.

The explanation approaches presented above in combination with the findings by Bohlmann et al. (2015) give rise to the supposition that EC plays an important role not only for first but also for second language development. First language competence is commonly acquired in familial contexts, which are characterized by smaller dyads (e.g., parent–child) and thus pose less demand on attentional regulation as a crucial part of EC. However, second language development commonly occurs in educational settings (e.g., preschools, daycare institutions; Winsler et al., 2008), associated with larger groups and hence more distractions, indicating a greater risk of interferences. Consequently, we hypothesize a stronger interrelation between EC and language competence in DLLs compared to monolinguals.

### Aims of the Present Study

The present study aims at evaluating the relation between EC and both receptive and expressive language competence in DLLs with German as a second language in preschool and kindergarten children. Current evidence of a link between EC and language is mainly based on studies with monolingual children (e.g., Dixon and Shore, 1997; Karrass and Braungart-Rieker, 2003; Salley and Dixon, 2007). The question of whether these findings are applicable to DLLs has rarely been addressed until now, yet it is postulated that interrelations between EC and language vary, depending on language acquisition groups. The present study thus pursues three specific aims. First, we investigate the relation between EC and concurrently assessed receptive and expressive language competence in preschoolers with German as their second language (DLLs exclusively). We hypothesize a significant interrelation between EC and language competence in preschoolers with DLL. Second, we investigate whether EC has a longitudinal effect on language competence in DLLs, when controlling for their initial level of language. We thus hypothesize that DLLs with higher EC show a more favorable second language development compared to DLLs with lower levels of EC. Third, we compare the longitudinal relationship between EC and language competence at the end of kindergarten between monolinguals and DLLs. We hypothesize that the relationship between EC and language competence is stronger in DLLs compared to monolinguals.

## Materials and Methods

### Procedure

The present study is based on data of the research project *^Zweit^Sprache* [English translation: ^Second^Language], which applies a prospective longitudinal design across early and middle childhood and was conducted in Basel, Switzerland starting in the year 2009. Its main aim was to evaluate the effect of early education on second language competence in immigrant children. The sample consists of four consecutive birth cohorts^1^. The sample comprised *n* = 429 children at the first assessment session (T_1_) and *n* = 242 children at the second assessment session (T_2_). The time lag between the first and the second assessment session was 16.52 months (*SD* = 0.64). The attrition rate is attributable to a structurally caused reduction of the sample^2^ and to a natural dropout of 23%. Children without a second assessment did not differ from children with complete sets of data in regard to age, sex, maternal education, EC, and in DLLs, all language subtests at T_1_ (all *p*s > 0.05).

Language tests were carried out by trained research assistants with a bachelor’s degree in psychology. The assessment of language tests was part of an assessment battery that lasted approximately 1.5 h and was conducted at the children’s homes. At the beginning of each assessment session, the research assistant started with a play session of around 10 min; this aimed to familiarize the child with the research assistant and the test situation. In a next step, the research assistant conducted the German language tests, followed by general development tests (e.g., emotion recognition). Socio-demographic and language characteristics were assessed by means of a parental questionnaire in the 10 most frequent migrant languages. Research assistants were accompanied by an intercultural intermediary (i.e., a translator) who explained the procedure of the assessment to the parents and explained the completion of the parental questionnaire in the event of insufficient parental local language competence. The questionnaire assessing EC was completed at around the time of the first language assessment by childcare or kindergarten teachers. The research project was approved by the Ethics Review Committee of the City and the County of Basel (EKBB) and conforms to the relevant regulatory standards.

### Participants

The sample consisted of *n* = 351 DLLs with German as a simultaneous (*n* = 40) or sequential (*n* = 311) second language (48% girls) and *n* = 78 monolingual German-speaking children (55% girls) at T_1_ and *n* = 172 DLLs and *n* = 70 monolinguals at T_2_. Classification to groups of monolinguals versus DLLs was obtained with parental questionnaires. Children were allocated to the monolingual group if German was indicated as their only used language and to the DLL group if any other language or multiple languages were mentioned as first language indicating that German was their second language. All kindergarten and early primary classes were taught exclusively in German (second language for DLLs) with no immersion classes.

Mean age at T_1_ was 4.85 years (*SD* = 0.31 years, age range 4.20–5.47 years) and 6.25 years (*SD* = 0.30 years) at T_2_. Socio-demographic characteristics classified by language groups (DLLs versus monolingual children) can be found in **Table 1**. The most frequent first languages in the subsample of DLLs were Turkish (16%), Italian (12%), Tamil (11%), English (10%), Spanish (8%), Albanian (8%), and Portuguese (6%). The group of DLLs consisted of children of 52 different nationalities: 28% Swiss, 11% Turkish, 9% Italian, 9% Sri Lankan, 4% Portuguese, 4% Serbian and Montenegrin, 3% German, 3% British, and 44 further countries.

**Table 1:** Comparison of sociodemographic and individual characteristics in dual language learners (DLLs) and monolingual children.

	DLLs with German as second language			Monolingual German-speaking children			Independent samples *t*-test between DLLs and monolinguals			
			
	*n*	Mean/ (%)	*SD*	*n*	Mean/ (%)	*SD*	*t*	*df*	*p*	*d*
Age in years (T_1_)	351	4.84	0.32	78	4.88	0.30	0.97	427	0.33	-0.13
Age in years (T_2_)	172	6.25	0.31	70	6.25	0.30	-0.18	240	0.86	0.00
Percentage female (T_1_)	351	(49.0)	–	78	(55.1)	–	1.21	427	0.23	–
Percentage female (T_2_)	172	(51.2)	–	70	(52.9)	–	0.24	240	0.81	
ME (T_1_)	351	3.66	1.28	78	4.19	0.97	3.96	427	<0.001	-0.43
ME (T_2_)	172	3.65	1.24	70	4.18	0.97	3.59	240	<0.001	-0.45
EC (T_1_)	351	4.76	1.11	78	5.19	0.95	2.48	427	0.02	-0.40
SETK-2										
WC (T_1_)	351	53.28	11.75	78	61.76	1.45	13.02	427	<0.001	-0.80
SC (T_1_)	351	54.77	11.21	78	63.73	2.98	13.02	427	<0.001	-0.88
WP (T_1_)	351	44.07	13.80	78	70.69	7.74	23.18	427	<0.001	-2.06
SP (T_1_)	351	46.23	17.81	78	71.71	13.52	14.11	427	<0.001	-1.49
SET 5–10										
LC (T_2_)	172	42.16	11.53	70	52.82	11.29	6.48	240	<0.001	-0.93
PiN (T_2_)	172	35.86	13.50	70	54.04	12.69	9.67	240	<0.001	-1.37
PiS (T_2_)	172	53.48	19.10	70	65.90	17.25	4.72	240	<0.001	-0.67
Global score (T_2_)	172	43.83	12.08	70	57.59	10.01	9.09	240	<0.001	-1.19


*ME, maternal education; EC, effortful control; WC, Word Comprehension; SC, Sentence Comprehension; WP, Word Production; SP, Sentence Production; LC, Language Comprehension; PiN, Picture Naming; PiS, Picture Story; analyses based on imputed data, estimates pooled.*

Eighty-one percent of the children of the DLL sample were born in Switzerland and 19% immigrated into Switzerland in their early years (*M* = 1.24, *SD* = 1.17 years). Mothers’ highest educational qualifications were assessed based on European categories for countries with a dual educational system. The categories were: 1 = no school education (3%), 2 = compulsory school (22%), 3 = apprenticeship or comparable (16%), 4 = academic high school or comparable (18%), and 5 = college or university (37%).

### Measures

#### Effortful Control

Effortful control was assessed using the German version of the *Child Behavior Questionnaire* (very short form; Putnam and Rothbart, 2006) by childcare and kindergarten teachers at T_1_. The Child Behavior Questionnaire is a widely used assessment of temperament used in early to middle childhood^3^. The present study used the subscale EC, consisting of 12 items (i.e., “*When drawing or coloring in a book, shows strong concentration*” and “*Is quickly aware of some new item in the room*”). Children’s EC ability was rated on a 7-point Likert scale ranging from *1 = extremely true* to *7 = extremely untrue*. In the current sample, internal consistency of the subscale was rated as good, with a Cronbach’s α = 0.84. Items were averaged to form an EC score.

#### Language

At T_1_, German language competence was measured with the standardized language development test SETK-2 (*Sprachentwicklungstest für zweijährige Kinder*; Grimm, 2000), which was designed for monolingual German-speaking children aged 2 years. The SETK-2 assesses children’s expressive and receptive vocabulary, as well as morphological and syntactical aspects of the German language. A pilot study indicated very low German language competence in DLLs (Keller, 2009). Therefore, the SETK-2 was applied despite a higher chronological age of the DLL sample compared to test norms. This procedure allowed us to prevent floor effects in DLLs with regards to their substantially lower competence spectrum. Although the SETK-2 was applied to both monolinguals and DLLs, for hypotheses testing, DLL children’s data were used exclusively due to ceiling effects in monolinguals.

The SETK-2 consists of four subtests: *Word Comprehension*, *Sentence Comprehension*, *Word Production*, and *Sentence Production*. Both language comprehension subtests of the SETK-2 are similar in structure to the Peabody Picture Vocabulary Test (Dunn and Dunn, 2007) where the child was presented four colored pictures from which the child had to choose the correct alternative form when orally presented a word or sentence. In both subtests of language production, children were presented with picture cards that depicted either objects or actions, which had to be named or described. The four subtests *Word Comprehension*, *Sentence Comprehension*, *Word Production*, and *Sentence Production* range from 0–8 points, 0–9 points, 0–24 points and 0–77 points, respectively. In the present study, standard values (*T*-scores) were used as dependent variables, based on the German monolingual 30- to 35-month-old norm sample (Grimm, 2000). In the current sample, internal consistencies were α_Word Comprehension_ = 0.85, α_Sentence Comprehension_ = 0.81, α_Word Production_ = 0.95, and α_Sentence Production_ = 0.93.

At T_2_, language competence was assessed in the entire sample with three subtests of the SET 5–10 (*Sprachstandserhebungstest für Kinder im Alter zwischen 5 und 10 Jahren*; Petermann, 2010), a language development test for children aged 5–10 years. In the present study, the subtests *Language Comprehension*, *Picture Naming*, and *Picture Story* were applied. The subtest Language Comprehension assesses the comprehension of complex sentence structures (main and subordinate clauses). Children were thus read 12 sentences that had to be replayed with toys. The subtest Picture Naming assesses expressive vocabulary. Children were presented 40 picture cards of objects and actions that had to be named (e.g., stamp, thermometer, or painting a wall). In the subtest Picture Story, children were asked to tell a story based on five consecutive pictures. This narrative was subsequently analyzed based on predefined semantic and syntactic criteria. The three subtests Language Comprehension, Picture Naming, and Picture Story range from 0–12 points, 0–40 points, and 0–8 points, respectively. However, for all further analyses, standard values of the SET 5–10 (*T*-scores) were used (Petermann, 2010). In the current sample, internal consistencies were α_LanguageComprehension_ = 0.94, α_PictureNaming_ = 0.80, and α_PictureStory_ = 0.74. In order to gain a broader verbal competence score, a global score was built, based on the average of the three subtests. The internal consistency of the global score was Cronbach’s α = 0.78.

### Data Analysis

Multiple imputation in SPSS was applied regarding missing data; this avoids substantial sample size reduction. Using all variables of the multiple imputation model obtains estimations of relations among variables, model parameters and standard errors, and has been proven to constitute a more accurate estimation of regression parameters than conventional methods such as mean substitution or listwise deletion (IBM, 2013). Missing values and subsequent imputations were reported in 3.4% of all cells. Five datasets were estimated. Estimates were pooled based on these five datasets. No imputations were obtained for T_2_ data of children who did not participate beyond T_1_.

## Results

### Descriptives

Dual language learners and monolingual children did not differ with respect to sex, age for T_1_ and for T_2_ (all *p*s > 0.05, see **Table 1**); however, differences were revealed in terms of maternal education, EC, and language competence at T_1_ and T_2_. Differences in the levels of education are in accordance with Swiss Federal Statistical Office data, reporting considerable differences in the level of education between native and immigrant populations (BFS, 2013). Moreover, as expected, significant group differences were found in all language tests. However, due to expected ceiling effects in language tests in monolinguals at T_1_, hypotheses 1 and 2 are limited to the DLL sample and hypothesis 3 used data at T_2_ exclusively.

### Effect of EC on Second Language Competence

The first hypothesis assessed whether the temperament dimension of EC was related to receptive and expressive second language competence. Based on data of the DLL subsample, hierarchical regression analyses were calculated with *Word Comprehension*, *Sentence Comprehension*, *Word Production*, and *Sentence Production* as dependent variables. Teacher-rated EC was applied as predictor; sex, age, and maternal education were applied as control variables. As can be seen in **Table 2**, significant interrelations between EC and both receptive as well as expressive language were found. The higher the estimation of children’s EC, the higher was the receptive and expressive second language competence found in the 1st year of kindergarten for Sentence Comprehension (β = 0.25), Word Production (β = 0.30), and Sentence Production (β = 0.23). However, EC explained only 5–8% of the variances in second language competence which are considered as small to medium effect sizes (Cohen, 1992).

**Table 2:** Effects of effortful control on second language competence at T_1_ (cross-sectional analyses, dual language learners, *n* = 351).

	Word comprehension				Sentence comprehension				Word production				Sentence production			
				
Predictors	Δ*R*^2^	β	*T*	*p*	Δ*R*^2^	β	*T*	*p*	Δ*R*^2^	β	*T*	*p*	Δ*R*^2^	β	*T*	*p*
Step 1	0.06			<0.001	0.12			<0.001	0.08			<0.001	0.09			<0.001
Constant			1.74	0.08			1.27	0.20			-0.73	0.46			-1.13	0.26
Sex^a^		0.03	0.47	0.64		0.00	0.08	0.93		0.02	0.47	0.64		0.02	0.48	0.63
Age		0.16	3.01	<0.01		0.13	3.78	<0.001		0.20	3.83	<0.001		0.18	3.41	<0.01
ME		0.20	3.79	<0.001		0.31	6.15	<0.001		0.24	4.68	<0.001		0.27	5.15	<0.001
Step 2	0.06			<0.01	0.06			<0.001	0.08			<0.001	0.05			<0.001
Constant			1.21	0.23			0.68	0.50			-1.46	0.15			-1.67	0.10
Sex^a^		-0.01	-0.09	0.93		-0.03	-0.56	0.58		-0.02	-0.30	0.77		0.01	-0.09	0.93
Age		0.12	2.00	0.05		0.15	2.63	0.01		0.15	2.77	0.01		0.14	2.55	0.01
ME		0.18	3.41	<0.01		0.29	5.88	<0.001		0.22	4.34	<0.001		0.25	4.88	<0.001
EC (T_1_)		0.24	2.32	0.05		0.25	2.73	0.03		0.30	4.61	<0.001		0.23	3.10	0.01
_corr_*R*^2^ (total)	0.11				0.18				0.17				0.13			


*ME, maternal education; EC, effortful control. ^a^Sex: 1 = male, 2 = female; analyses based on imputed data, estimates pooled.*

Hypothesis 2 examined whether higher EC was associated with a more favorable second language *development*. Therefore, four longitudinal regression analyses were carried out for DLLs, including children’s initial level of receptive and expressive language competence at T_1_^4^ , predicting second language competence at T_2_. Dependent variables were the three subtests Language Comprehension, Picture Naming, and Picture Story, plus the global score of the SET 5–10. Sex, age, time lag between T_1_ and T_2_, maternal education, and, depending on the target variable, initial levels of either receptive or expressive language competence served as control variables. Time lag and initial levels of language competence were included as additional control variables as Hypothesis 2 aims at depicting developmental progression. We therefore intended to avoid that differences may be explained by initial differences in language competence or developmental differences due to different time lags between T1 and T2. Results indicate a more favorable second language development in children with higher levels of EC (see **Table 3**). Small yet significant effects of EC were found in the subtests Language Comprehension and Picture Naming, and the global score of the SET 5–10 (explained variance: 1–4%). Only tendentially significant results were found for the subtest of Picture Story.

**Table 3:** Effects of effortful control on second language competence at T_2_ (longitudinal analyses, dual language learners, *n* = 172).

	Language Comprehension (T_2_)				Picture Naming (T_2_)				Picture Story (T_2_)				Global score (T_2_)			
				
Predictors	Δ*R*^2^	β	*T*	*p*	Δ*R*^2^	β	*T*	*p*	Δ*R*^2^	β	*T*	*p*	Δ*R*^2^	β	*T*	*p*
Step 1	0.24			<0.001	0.58			<0.001	0.25			<0.001	0.53			<0.001
Constant			0.93	0.35			2.57	0.01			1.24	0.21			2.11	0.04
Sex^a^		0.00	0.06	0.96		-0.09	-1.78	0.08		-0.12	-1.72	0.09		-0.10	-1.82	0.07
Age		-0.19	-2.69	<0.01		-0.32	-6.01	<0.001		-0.02	-0.33	0.74		-0.22	-3.83	<0.001
Time lag		0.11	1.54	0.12		0.11	2.19	0.03		-0.02	-0.25	0.80		0.08	1.42	0.15
ME		0.14	1.97	0.05		0.00	0.08	0.94		-0.09	-1.32	0.19		-0.02	-0.40	0.69
Comp (T_1_)		0.42	5.95	<0.001		–	–	–		–	–	–		0.11	1.33	0.19
Prod (T_1_)		–	–	–		0.76	14.23	<0.001		0.50		<0.001		0.67	8.45	<0.001
Step 2	0.04			0.02	0.01			0.02	0.03			0.06	0.03			0.01
Constant			0.77	0.44			2.37	0.02			1.05	0.29			1.88	0.06
Sex^a^		-0.02	-0.28	0.78		-0.10	-2.04	0.04		-0.13	-1.97	0.05		-0.12	-2.15	0.03
Age		-0.20	-2.85	0.00		-0.33	-6.13	<0.001		-0.03	-0.38	0.70		-0.22	-3.94	<0.001
Time lag		0.10	1.50	0.13		0.11	2.15	0.03		-0.02	-0.32	0.75		0.07	1.34	0.18
ME		0.13	1.89	0.06		0.00	0.05	0.96		-0.10	-1.39	0.17		-0.03	-0.45	0.65
Comp (T_1_)		0.38	5.12	<0.001		–	–	–		–	–	–		0.10	1.19	0.24
Prod (T_1_)		–	–	–		0.72	12.34	<0.001		0.46	5.99	<0.001		0.62	7.26	<0.001
EC		0.20	2.15	0.04		0.13	2.19	0.03		0.16	1.80	0.08		0.17	2.40	0.02
_corr_*R*^2^ (total)	0.25				0.58				0.25				0.54			


*Time lag, time lag between T_*1*_ and T_*2*_; ME, maternal education; Comp, mean of language subtests Word Comprehension and Sentence Comprehension; Prod, mean of language subtests Word Production and Sentence Production; EC, effortful control. ^a^Sex: 1 = male, 2 = female; analyses based on imputed data, estimates pooled.*

To examine the practical relevance of EC in second language competence, a further analysis was conducted comparing the quartile of DLLs with lowest levels of EC with the quartile of DLLs with highest levels of EC. Difference in second language competence in children with low versus children with high EC expressed in months were calculated based on the age-norms of the three subtests Language Comprehension, Picture Naming, Picture Story provided by the SET 5–10 Manual (Petermann, 2010). The analysis revealed that DLLs with the lowest levels of EC showed a discrepancy in second language competence equivalent to a developmental difference of more than 14 months at the end of kindergarten compared to DLLS with the highest levels of EC (second language competence of DLLs at T_2_, first quartile of EC, *M* = 37.2; fourth quartile of EC, *M* = 48.9).

### Comparison between DLLs and Monolinguals

**FIGURE 1:**
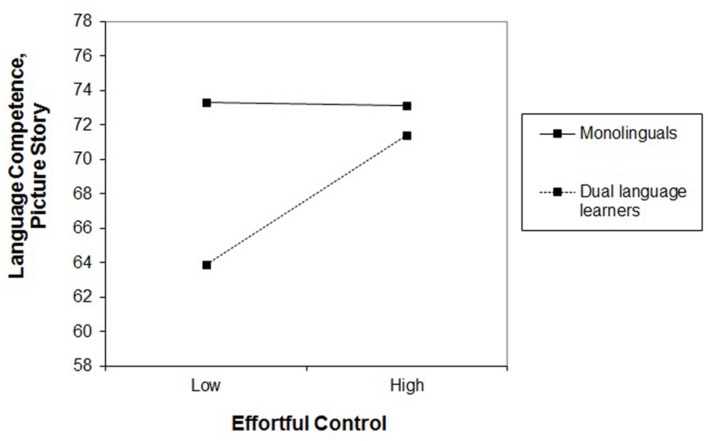
**Interaction plot of language group and effortful control on the subtest Picture Story at T_2_**.

The third hypothesis tested whether the longitudinal effect of EC on second language competence in DLLs was stronger than the effect of EC on first language competence in monolingual children. In order to statistically compare the effects between the two groups, four moderation analyses were conducted with the complete sample. The three language subtests and the global score at T_2_ served as dependent variables. In a first step, the control variables sex, age, maternal education, language group (DLLs versus monolinguals) as well as centered values of EC were entered as predictors and in a second step the interaction term group × EC. Analyses revealed no significant interactions for the subtests Language Comprehension and Picture Naming. However, effects of EC were significantly larger on the subtest Picture Story and tendentially significantly on the global score in the group of DLLs, compared to the monolingual German-speaking group (**Table 4**). In DLLs, EC was related to narrative competence with β = 0.29 (*p* < 0.01) and β = 0.39 (*p* < 0.001) for language competence as measured by the global score whereas in monolinguals, EC was related to narrative competence with β = -0.14 (*p* = 0.32) and β = 0.12 (*p* = 0.38) for global language competence. These findings suggest that language competence and in particular narrative competence in DLLs seem to depend on EC more markedly than language competence in monolingual children (see **Figure 1**). However, the interaction terms only explained 4% of the variance in the subtest Picture Story and 2% in the global score, which signify small effects (Cohen, 1992).

**Table 4:** Comparison between dual language learners and monolinguals at T_2_ (longitudinal analyses, *n* = 242).

	Language Comprehension (T_2_)				Picture Naming (T_2_)				Picture Story (T_2_)				Global score (T_2_)			
				
Predictors	Δ*R*^2^	β	*T*	*p*	Δ*R*^2^	β	*T*	*p*	Δ*R*^2^	β	*T*	*p*	Δ*R*^2^	β	*T*	*p*
Step 1	0.26			<0.001	0.40			<0.001	0.14			<0.001	0.32			<0.001
Constant			5.14	<0.001			5.13	<0.001			1.14	0.26			4.23	0.02
Sex^a^		-0.06	-0.98	0.33		-0.10	-1.82	0.07		-0.15	-2.46	0.01		-0.13	-2.34	0.19
Age		-0.17	-2.69	<0.01		-0.18	-3.11	<0.01		0.09	1.46	0.14		-0.08	-1.32	0.06
ME		0.15	2.49	0.01		0.13	2.23	0.02		0.03	0.44	0.66		0.11	1.90	<0.001
Group^b^		-0.31	-4.96	<0.001		-0.45	-7.74	<0.001		-0.26	-4.02	<0.001		-0.40	-6.73	<0.001
EC		0.23	2.42	0.04		0.28	2.72			0.16	1.93	0.07		0.26	2.64	0.06
Step 2	0.01			0.36	0.00			0.52	0.04			<0.01	0.02			0.05
Constant			5.27	<0.001			5.23	<0.001			1.26	0.21			4.50	<0.001
Sex^a^		-0.06	-1.01	0.32		-0.10	-1.84	0.07		-0.15	-2.47	0.01		-0.13	-2.38	0.02
Age		-0.17	-2.77	0.01		-0.18	-3.16	<0.01		0.09	1.43	0.15		-0.08	-1.41	0.16
ME		0.15	2.45	0.011		0.13	2.27	0.02		0.02	0.38	0.70		0.11	1.88	0.06
Group^b^		-0.32	-5.00	<0.001		-0.45	-7.79	<0.001		-0.29	-4.57	<0.001		-0.42	-7.37	<0.001
EC		0.25	3.39	0.00		0.29	3.47	<0.01		0.19	2.37	0.02		0.29	4.11	<0.001
Group × EC		0.08	0.86	0.40		0.04	0.54	0.59		0.21	2.85	<0.01		0.15	1.95	0.06
_corr_*R*^2^ (total)	0.25				0.39				0.16				0.33			


*ME, maternal education; EC, effortful control. ^a^Sex: 1 = male, 2 = female; ^b^group: -1 = monolinguals, 1 = dual language learners; analyses based on imputed data, estimates pooled.*

## Discussion

This study aimed at assessing the interrelation between EC and second language acquisition in DLLs in kindergarten. Moreover, we evaluated whether there was a difference between the strength of the interrelation between EC and the second language of DLLs, and EC and the first language of monolinguals.

### Effect of EC on Second Language Competence

The assumption of a positive interrelation between EC and second language competence was confirmed. Both cross-sectional and longitudinal analyses revealed that children with higher EC display higher receptive and expressive second language competence. Although effects being small in size, our results indicate that EC is important not only in first language development (e.g., Blair and Razza, 2007; Salley and Dixon, 2007) but also in early second language development.

Most previous studies targeting the relation between EC and language are based on either concurrent or longitudinal correlations (e.g., Dixon and Shore, 1997; Dixon and Smith, 2000; Morales et al., 2000; Blair and Razza, 2007; Salley and Dixon, 2007). Consequently, no conclusions can be drawn regarding the effect of EC on language *development*. A developmental approach can be found in the studies by Slomkowski et al. (1992) and Karrass and Braungart-Rieker (2003) with monolinguals; however, in both studies the effects on language competence disappeared when controlling for initial levels of language. The present study also addressed the effect of EC on language development, controlling for language competence at T_1_ as well as sex, age, maternal education, and time lag between T_1_ and T_2_ in a longitudinal analysis with DLLs. Results revealed that EC, assessed at age 5 years, was a significant positive predictor of subsequent second language competence. Moreover, kindergarten children with lower EC showed a higher probability of less favorable second language development than children with higher EC. The practical relevance of this finding becomes evident by comparing groups with low versus high levels of EC with regard to second language competence. Comparing the quartile of DLLs with lowest levels of EC with the quartile of children with highest levels of EC reveals a discrepancy in second language competence equivalent to a developmental difference of more than 14 months at the end of kindergarten. Given that such an accentuated developmental delay is difficult to compensate and may even enlarge across compulsory school years, DLLs with low EC are at risk of language deficits and an unfavorable school career.

Our results are in contrast to previous findings in preschool children (Slomkowski et al., 1992; Karrass and Braungart-Rieker, 2003): whereas in the studies by Slomkowski et al. (1992) and Karrass and Braungart-Rieker (2003), the positive effects of EC on language competence disappeared controlling for initial levels of language, the results of the present study reveal that EC predicts second language development. One possible reason for diverging findings may be due to differences regarding children’s age ranges and thus related developmental stages of age-normative development of EC. According to Rothbart and Rueda (2005), children experience considerable development in EC between 2 and 7 years of age. In this age range, the influence of EC on language development may be particularly obvious due to increased individual differences in developmental trajectories of EC. As children in the present sample were substantially older than in the studies by Karrass and Braungart-Rieker (2003; 12- to 16-month-old children) and Slomkowski et al. (1992; 2- to 3-year-old children), a closer link between EC and language could therefore be explained with a more advanced EC development. Moreover, in the current study, DLLs were examined whereas in the studies by Slomkowski et al. (1992) and Karrass and Braungart-Rieker (2003) monolinguals were investigated. Thus, another reason for the diverging findings might be due to the bilingual status of the different samples. This will be discussed in more detail in Section “Comparison Between DLLs and Monolinguals.”

The literature traces the relation between EC and language competence mainly on two mechanisms: social interactions and attentional processes. On the one hand, we assume that higher self-regulatory abilities are associated with more frequent and qualitatively more favorable interactions with peers and teachers not only in monolinguals (Eisenberg et al., 2007; Valiente et al., 2008), but also in the scope of the development of second language competence. Interaction processes may constitute an important resource particularly for DLLs who are mainly or exclusively surrounded by their language of origin in their familial contexts. Moreover, natural communication situations with peers or teachers are of particular importance in early childhood when the development of second language competence ensues mainly without guidance and explicit instructions (Gogolin, 2009). On the other hand, we assume that attentional processes are relevant not only for the development of first language competence (Dixon and Smith, 2000; Dixon et al., 2006), but in particular in the scope of the development of second language competence given the diverging contexts of first- versus second language acquisition (Winsler et al., 2008; Becker, 2010; Hoff, 2013). As studies have shown (e.g., Winsler et al., 2008; Becker, 2010), the extrafamilial context is more important for local language learning for DLLs than for monolinguals. In extrafamilial contexts activities are often undertaken in group settings, associated with larger groups and hence more distractions, indicating a greater risk of interferences. Effective protection against distractions and concentration on relevant task demands are generally important prerequisites for learning (e.g., Blair and Razza, 2007). Vocabulary development in particular, displaying substantial developmental changes in kindergarten age, requires an efficient word-referent mapping, which is essentially associated with attentional control (Dixon et al., 2006).

### Comparison between DLLs and Monolinguals

In addition, we examined whether the effect of EC on language competence was larger in DLLs compared to monolingual children. Inferential statistical comparisons between both groups revealed, as postulated, a significant interaction effect in favor of DLLs. These findings indicate that EC seems to be a more important predictor of general language competence and narrative abilities in DLLs compared to monolingual children. In the other language subtests, however, group differences did not reach a level of significance. The hypothesis of specific effects depending on language groups (monolinguals versus DLLs) can thus be evaluated as partially confirmed. One reason for the differential effects between DLLs and monolingual children may be related to the *context* of the development of language competence. Whereas early first language development is mainly based on dyadic parent–child interactions, early educational institutions seem to be of particular importance for the early development of second language competence (Keller et al., 2015). Due to their group size and structure, however, they place high demands on children’s EC. In this way, the development of second language competence may potentially be more vulnerable to unfavorable EC than first language development because the development of second language competence is closely dependent on these extra-familial contexts.

The results of the present study, varying substantially depending on language tasks, prompted the question of whether the mastery of different language tasks requires different components of EC. It can be assumed that tasks that are particularly multifaceted (such as the narrative of a picture story that comprises discursive, metalinguistic, semantic, and morphosyntactic competence) entail higher demands on EC and attentional reactivity, among other higher-order processes. This complexity could easily be an excessive demand for DLLs with low EC who are already sufficiently challenged by the learning setting of early educational institutions.

### Limitations and Future Directions

The present study contributes to the scarcely studied field of literature aims to assess the effect of EC on the early development of second language competence. Results highlight the importance of children’s individual characteristics for their language development. Moreover, it is noteworthy that the applied longitudinal design, with a period of 16 months between assessment sessions and the consideration of several central control variables, allowed statements about the predictive power of EC on second language development for the first time.

Even though the present study provided evidence of EC as a small yet significant factor in the early development of second language competence, there are some limitations and several starting points for further differentiating these findings in future studies.

First, it would be desirable to explore potentially mediating factors between EC and second language. It can be assumed that there are multiple mechanisms of action comprising both social interactions as well as attentional processes. In particular, with regard to early interventions it would be of central importance to evaluate these mediating processes more precisely. Moreover, it would be interesting to analyze whether the explanatory mechanisms underlying the development of first and second language competence are comparable.

Second, a more comprehensive battery for the assessment of EC in future studies, allowing statements about different components of EC, would be favorable. In their assessment of monolingual children, Salley and Dixon (2007) demonstrated that in particular inhibitory control and attentional components, assessed through parental reports showed considerable interrelations with language, while smaller effects on language were found for low-intensity pleasure and affiliation. Whether these patterns of results could be confirmed in samples of different ages and in DLLs could be the subject of further studies.

Third, we did not assess German language competence in monolingual and bilingual children at age 4–5 years with an age-normed instrument. At the time the project *^Zweit^Sprache* was started, no instrument was available that was validated, provided age norms for both monolinguals and bilinguals and that also captured different German language aspects. However, a pilot study revealed that the SETK-2 was appropriate for bilingual children (Keller, 2009), who were the focus of the project *^Zweit^Sprache*. Using the SETK-2 at T_2_, we were limited in comparing the language test scores and language development of bilingual and German monolingual children because of a floor effect in monolingual children. It would be desirable for future studies to develop and use age-normed measures that are appropriate for both monolingual and bilingual children at an early and assess language competence for a larger developmental range.

Fourth, it would be important to assess the effect of EC on DLLs’ *first* language. The present study compared the effect of EC on first language competence of monolingual children with the effect of EC on second language competence of DLLs. The additional comparison of the effect of EC on DLLs’ first language would provide information whether these stronger effects in DLLs are attributable to the development of second (versus first) language competence, or rather bilingualism (versus monolingualism). This question remains unanswered in the present study due to the confounding of these two dimensions.

### Implication for Education and Conclusion

With regard to an improvement in second language competence, one approach might be to raise the educational authorities’ awareness of the learning requirements of DLL children with low EC and to broaden the pedagogical repertoire of kindergarten and primary school teachers in order to specifically support these children. To ensure that early educational institutions have an adequate structural quality, both an appropriate class size and a suitable location allowing quiet as well as physically active school activities are of utmost importance (OECD, 2011). Moreover, pedagogic strategies such as the purposeful sequencing of the teaching units and the minimizing of interfering stimuli are particularly central in language learning sequences in the development of second language competence (Iaquinta, 2006; Copple and Bredekamp, 2009).

Local language competence has been shown to be crucial in order to master the transition from kindergarten to school and to succeed in school (Merz et al., 2014). It is of utmost importance to evaluate factors determining the acquisition of local language competence and thereby contribute to the reduction of inequalities of opportunities for DLLs (De Feyter and Winsler, 2009; Winsler et al., 2014). Even though the promotion of early second language competence has recently gained increasing attention in educational policy, knowledge about the development of second language competence is still scarce (Hoff, 2013). The findings of the present study highlight the importance of DLL children’s EC as a significant determinant of second language competence that should be particularly focused on by affirmative actions.

## Author Contributions

The manuscript is based on the research ideas of KK and her analyses. However, all authors made substantial contributions to the conception of the manuscript and the interpretation of the data. Moreover, all authors have approved the manuscript and agree with its submission in the current form.

## Conflict of Interest Statement

The authors declare that the research was conducted in the absence of any commercial or financial relationships that could be construed as a potential conflict of interest.

## Footnotes

1 No significant differences were found between all four cohorts in respect of sex, time lag between assessment sessions, and maternal education (all *p*s > 0.05); data of all cohorts were consequently combined to one sample.

2 For T_2_, a subsample of 314 out of the original 429 families was invited to participate, of which 242 families responded and consented to participate in the assessment.

3 http://www.bowdoin.edu/sputnam/rothbart-temperament-questionnaires/

4 For the bilingual sample of the current study, the internal consistencies were Cronbach’s α = 0.81 for Language Comprehension and Cronbach’s α = 0.91 for language production.
